# Surgical treatment of chronic acromioclavicular joint dislocation with autogenous tendon grafts

**DOI:** 10.1186/2193-1801-3-420

**Published:** 2014-08-10

**Authors:** Kaisa J Virtanen, Vesa Savolainen, Ilkka Tulikoura, Ville Remes, Ville Haapamäki, Jarkko Pajarinen, Jan-Magnus Björkenheim, Mika Paavola

**Affiliations:** Department of Surgery, Helsinki University Central Hospital and University of Helsinki, Topeliuksenkatu 5, Helsinki, 00260 Finland; Department of Radiology, Helsinki Medical Imaging Centre, Helsinki University Central Hospital, Topeliuksenkatu 5, Helsinki, 00260 Finland; Centre for Health and Social Economics, Institute for Health and Welfare, PL 30, Helsinki, 00271 Finland

**Keywords:** Chronic acromioclavicular joint dislocation, Delayed surgical treatment, Tendon graft

## Abstract

**Background:**

Conservative treatment of acromioclavicular (AC) joint dislocation is not always successful. A consequence of persistent AC joint dislocation may be chronic pain and discomfort in the shoulder region as well a sensation of constant AC joint instability and impaired shoulder function. Stabilization of the AC joint may reduce these sequels.

**Materials and methods:**

Due to chronic AC joint dislocation, 39 patients in our hospital underwent coracoclavicular (CC) ligament reconstruction with autogenous semitendinosus and gracilis tendons between May 2005 and April 2011. We examined 25 patients after a mean of 4.2 years. The outcomes were Constant shoulder Score (CS), Disabilities of the Arm, Shoulder and Hand (DASH), pain (Visual Analog Scale, VAS), cross-arm test, stability of the AC joint, and complications. The follow-up visits included anteroposterior and axillary radiographs.

**Results:**

Mean CS was 83 in the injured shoulder and 91 in the uninjured shoulder (p = 0.002). Mean DASH was 14. In 14 patients, the AC joint was clinically stable; pain was minor. In radiographs, osteolysis of the lateral clavicle and tunnel widening were markedly common. Fracture of the coracoid process occurred in 5 patients, and 3 suffered a fracture of the clavicle; 2 had a postoperative infection.

**Conclusions:**

Anatomic reconstruction of CC ligaments showed a moderate subjective outcome at the 4-year follow-up. After surgery, almost half the AC joints failed to stabilize. Lateral clavicle osteolysis and tunnel widening were notably common complications.

## Background

Acromioclavicular (AC) joint dislocations are typically classified according to Rockwood, a classification based on degree of soft-tissue injury and clavicle dislocation (Galatz et al. 2009). Clinical assessment of the severity of the AC joint injury is challenging. It is not uncommon that the severity of the injury is misclassified. Sometimes, result of conservative treatment is unsatisfactory. The patient may suffer chronic pain and discomfort in the shoulder region, as well impaired shoulder function (Bannister et al. 1989; Kennedy and Cameron 1954; Song et al. 2012).

To date, the literature abounds in different methods to treat chronic AC joint dislocation (Adam and Farouk 2004; Bostrom Windhamre et al. 2010; Fraschini et al. 2010; Jeon et al. 2007; Kim et al. 2012; Tauber et al. 2009). No one of these methods seems superior. Anatomic reconstruction of coracoclavicular (CC) ligaments with autogenous tendon grafts, widely used in treating chronic AC joint instability, reportedly diminishes pain, eliminates sequels, and improves function as well as strength (Jones et al. 2001; LaPrade and Hilger 2005; Shetty et al. 2009; Tauber et al. 2009; Yoo et al. 2010; Tauber et al. 2007). Semitendinosus and gracilis tendons are evaluated to be strong enough to use as suitable grafts (Hamner et al. 1999).

The aim of our study was to assess the functional and radiological results in chronic AC joint dislocation treated with autogenous semitendinosus and gracilis tendon grafts. Our hypothesis was that results of delayed surgery are satisfactory, but function is inferior to the function of the uninjured side. Before the study began, Helsinki University Central Hospital, Ethics Committee, Department of Surgery gave their approval (2004-02-27 66/E6/04). Patients approved the informed consent to participate in the study.

## Materials and methods

In our hospital, between May 2005 and April 2011, 39 patients had surgery for chronic AC joint dislocation. Of 39 patients, 25 participated in a follow-up visit after a mean of 4.2 years (range 1–7 years). The rest 14 patients refused the follow-up visit or did not respond to the invitation. Patient and injury characteristics are shown in Table 1. Indications for delayed surgery were long-term pain or discomfort in the shoulder region and inability to do normal work or daily tasks after unsuccessful conservative treatment (19 patients) or failure of an earlier surgery with persistent pain (6 patients). Mean delay from injury to reconstructive surgery was 435 (range 149–1586) days. In primary radiographs, the majority (15 patients) had Rockwood type V injury.

**Table 1:** **Patient and injury characteristics in 25 patients treated surgically for chronic AC joint dislocation with autogenous semitendinosus and gracilis tendon grafts**

Male/female	21/4
Injured side	
Right/left	18/7
Dominance	
Right/left	21/4
Mechanism of primary injury	
Bicycling	9
Simple fall	7
Sport	7
Traffic accident	2
Rockwood type in primary radiographs	
II	3
III	6
IV	1
V	15
Mean age at time of injury, y (range)	44 (22–59)
Mean age at time of examination, y (range)	48 (24–63)

### Surgical technique

Surgery was performed under general anesthesia by one surgeon. Clindamycin 600 mg antibiotic prophylaxis was administered to each patient, who lay in a standard, beach-chair position. After installing a tourniquet in the thigh, a vertical incision was made over the pes anserinus area. Semitendinosus (ST) and gracilis (G) tendons were identified and harvested by a tendon stripper. The wound was closed in layers. A saber incision was then made over the AC joint and distal clavicle. The AC joint and distal clavicle was exposed by detaching the origin of the deltoid muscle and the insertion of the trapezoid muscle. The coracoid process was exposed by excising the scar tissue and fat pad in the region of the CC ligaments. If a visible osteoarthritis existed in the AC joint, a 6–8 mm resection of the distal clavicle was performed with an oscillating saw. At the insertion sites of the CC ligaments in the clavicle were drilled 2 superior-inferior directed 5.5-mm holes. The prepared tendons and 2 FiberWire^®^ #5 (Arthrex, Naples, FL, USA) sutures were transferred under the coracoid process. After the AC joint was reduced manually, the tendons, once pulled through the drill holes, were then attached by 5.5 × 15-mm tenodesis screws (Bio-Tenodesis™ AR-1555B or PEEK Tenodesis™ AR-1555PS, Arthrex, Naples, FL, USA). If the tendon graft was sufficient over the AC joint, it was attached to the acromion with 3.5-mm titanium suture anchor (Corkscrew^®^, Arthrex) to strengthen the superior AC ligament. Finally, the double FiberWire^®^ #5 sutures (18 patients) were tied over the clavicle. The wound was closed in layers. The arm was immobilized in a sling for 2 to 3 weeks. Postoperatively, pendulum motion was allowed as well as passive abduction and flexion up to the horizontal plane. After 4 weeks the full range of motion was allowed without weight. After 6 months, patients could return to full activity. Detailed information on the surgery is in Table 2.

**Table 2:** **Description of surgery in the 25 patients treated for chronic AC joint dislocation**

Detail	n = 25
Resection of lateral end of clavicle	10
PEEK Tenodesis™ screws (5.5 × 15 mm)	8
Bio-Tenodesis™ screws (5.5 × 15 mm)	17
Strengthening of superior AC ligament	18
FiberWire^®^ (#5) augmentation	18
Semitendinosus graft	25
Gracilis graft	21
	**Minutes**
Mean duration of surgery* (range)	112 (71–180)

AC = acromioclavicular.

PEEK = polyetheretherketone.

*Duration with tendon harvesting.

The outcomes were Constant shoulder Score (CS), Disabilities of the Arm, Shoulder and Hand (DASH), pain (Visual Analog Scale, VAS 0–10), cross-arm test, clinical stability of the AC joint, and complications. Length of sick leave was determined, as was the patient’s subjective satisfaction with the end results of the treatment (excellent, good, moderate, poor). From anteroposterior (AP) and axillary radiographs, we evaluated alignment of the AC joint, AC joint osteoarthritis, osteolysis of the lateral clavicle, and possible complications. The surgery and clinical examination was performed by separate persons.

### Statistcs

Statistical analysis was performed with SPSS Statistics (version 21.0, IBM, USA). We used the Kolmogorov-Smirnov test for normality. The CS between the injured and uninjured shoulder was analyzed by paired-samples T-test. Nominal variables were analyzed by Fisher’s exact test. For all tests a 2-sided level of 0.05 was considered significant.

## Results

The mean CS was 83 (range 55–100) for the injured shoulder and 91 (range 77–100) for the uninjured shoulder (p = 0.002). The mean DASH was 14 (range 0–58). In 14 patients, the AC joint was clinically stable at follow-up. On the injured side, the cross-arm test was positive in 6 patients (Table 3). Mean length of sick leave after the reconstructive surgery was 109 (range 28–374) days. Of 25 patients, 21 assessed the subjective results as excellent or good.

**Table 3:** **Clinical outcomes after a mean 4.2 years in the 25 patients treated surgically for chronic AC joint dislocation**

	n = 25
CS injured shoulder	83 (12)
CS uninjured shoulder	91 (7)
DASH	14 (18)
VAS at rest (0–10)	1 (2)
VAS at activity (0–10)	3 (3)
Cross-arm test positive	6
AC joint clinically stable	14

AC = acromioclavicular.

CS = Constant shoulder Score.

DASH = Disabilities of the Arm, Shoulder and Hand.

VAS = Visual Analog Scale.

Standard deviation (SD) in parentheses.

**Figure 1:**
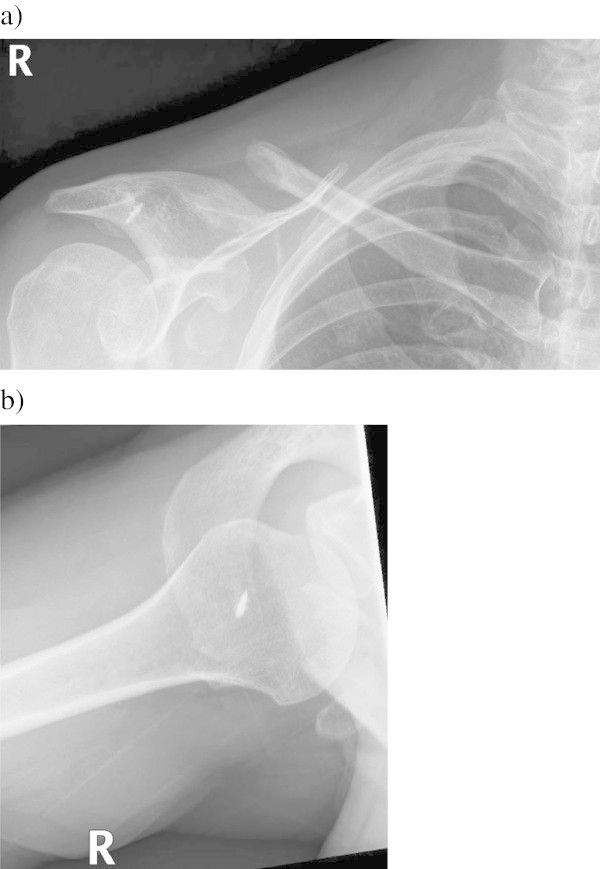
**Anteroposterior (a) and axillary (b) radiographs of a patient who had surgery for chronic AC joint dislocation with autogenous semitendinosus and gracilis tendon grafts 4 years previously.** Marked osteolysis has developed to the lateral clavicle. This patient also suffered a fracture of the coracoid process.

**Figure 2:**
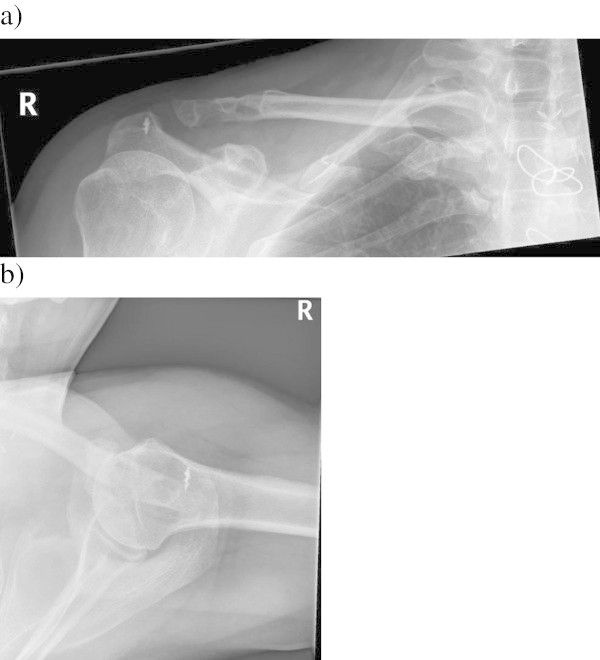
**Anteroposterior (a) and axillary (b) radiographs of a patient after surgery for chronic AC joint dislocation.** The injury was treated with autogenous semitendinosus and gracilis tendon grafts 5 years before. Notable tunnel widening has appeared in both drill holes. Erosion of the coracoid process is also visible.

In the radiographs (AP and axillary) the AC joint was in normal alignment in 11 patients. After the procedure, lateral clavicle osteolysis (Figure 1) and tunnel widening (Figure 2) were fairly common (Table 4). In only 1 patient the osteolysis was seen in the preoperative radiographs. We found no relation between screw material and osteolysis (p = 0.39) or tunnel widening (p = 0.28). Distal clavicle resection seemed not to influence to the incidence of osteolysis (p = 0.4).

**Table 4:** **Radiologic results after a mean 4.2 years in the 25 patients treated surgically for chronic AC joint dislocation**

	n = 25
Normal alignment of AC joint in AP projection	13
Normal alignment of AC joint in axillary projection	19
Normal alignment of AC joint in AP and axillary projections	11
AC osteoarthritis	1
Lateral clavicle osteolysis	14
Tunnel widening	20
Heterotopic ossification	2

AC = acromioclavicular.

AP = anteroposterior.

Postoperatively, 1 patient had a superficial and 1 patient a deep wound infection. The former was treated with p.o. antibiotics and the latter with i.v. and p.o. antibiotics. The deep wound infection resulted in removal of tendon grafts and screws and healed only after a deltoid-muscle transfer. In 2 patients, reconstructive surgery failed, and these required another identical procedure. Fracture of the coracoid process appeared in 5 patients, and 3 patients had a fracture in the clavicle (Table 5).

**Table 5:** **Clinical and radiological complications after a mean 4.2 years follow-up in the 25 patients treated surgically for chronic AC joint dislocation**

	n = 25
Wound infection	2
Clinical instability of AC joint	11
Tunnel widening	20
Lateral clavicle osteolysis	14
Incongruency of AC joint (in AP or in axillary radiograph)	14
Fracture of clavicle	3
Fracture of coracoid process	5
Reoperation	4

AC = acromioclavicular.

AP = anteroposterior.

In 18 patients, the superior AC ligament was reinforced with a tendon graft. Despite this procedure, in 7 patients their AC joint was unstable. In comparison, of 7 patients without the AC ligament reinforcement, 3 had an unstable AC joint. There was no difference between patients with clinically stable AC joint and unstable AC joint in function (p = 0.7), in disability (p = 0.6), or in patient satisfaction (p = 0.4).

We assessed the influence of osteolysis, tunnel widening, and fracture of the coracoid process on function, disability, pain, and AC joint stability. Patients having lateral clavicle osteolysis seemed to have more disability (DASH 19, range 0–58) than patients without it (DASH 8, range 0–28), although this difference was not statistically significant (p = 0.2) (Table 6).

**Table 6:** **Influence of osteolysis, tunnel widening, and fracture of the coracoid process on function (CS), disability (DASH), pain (VAS), and AC joint stability in patients treated with autogenous semitendinosus and gracilis tendon grafts for chronic AC joint dislocation**

	CS injured	CS uninjured	DASH	VAS at rest	VAS at activity	AC joint stable (n)
Osteolysis + (n = 14)	78	86	19	2	4	6
Osteolysis - (n = 11)	88	91	8	1	2	8
**p value**	**0.09**	**0.5**	**0.2**	**0.5**	**0.2**	**0.2**
Tunnel widening + (n = 20)	83	88	15	1	3	11
Tunnel widening - (n = 5)	83	88	11	1	3	3
**p value**	**1.0**	**0.5**	**0.8**	**0.7**	**0.9**	**1.0**
Coracoid fracture + (n = 5)	82	92	12	1	3	4
Coracoid fracture - (n = 20)	83	88	15	1	3	10
**p value**	**0.9**	**0.7**	**0.9**	**0.5**	**0.9**	**0.3**

CS = Constant shoulder Score.

DASH = Disabilities of the Arm, Shoulder, and Hand.

VAS = Visual Analog Scale (0–10).

AC = acromioclavicular.

+ = phenomenon exists.

- = phenomenon does not exist.

Results are after a mean 4.2-year follow-up.

## Discussion

Sometimes conservative treatment of AC joint dislocation is unsuccessful, or results of surgery are undesirable. Consequences may be persistent pain and insufficiency in the shoulder region, inability to perform overhead activities, and a repeated sense of instability or weakness. In our study, all patients were treated with anatomic reconstruction of the CC ligaments. In most patients, the superior AC ligament was also reinforced. At follow-up, clinical outcomes were moderate. The rate of complications was, however, surprisingly high.

In 1972, Weaver and Dunn published a method to treat chronic AC joint dislocation. The original procedure involved a resection to the lateral end of the clavicle and stabilization of the AC joint by the acromial end of the shortened coraco-acromial ligament into the medullary canal of the lateral clavicle (Weaver and Dunn 1972). During later decades, several modifications have arisen from the Weaver-Dunn method (Adam and Farouk 2004; Boileau et al. 2010; Bostrom Windhamre et al. 2010; Hosseini et al. 2009; Kim et al. 2012; Lafosse et al. 2005; Millett et al. 2009; Pavlik et al. 2001). Jones et al. (2001) first published a case report discussing reconstruction of CC ligaments by autogenous semitendinosus tendon graft. In chronic AC joint dislocation, earlier studies have evaluated the results of this normal-anatomy-imitating technique (Tauber et al. 2007; Tauber et al. 2009; Yoo et al. 2010; Fauci et al. 2013). These studies have suggested favorable results after surgery.

After the reconstructive surgery, we found satisfactory results for upper limb function and symptoms. CS was better in the uninjured than in the injured shoulder, but we believe that the difference (8 points) is not clinically relevant. According to the literature, the normal value of CS in this age group (40–50) is 91 to 96 in male and 83 to 86 in female (Katolik et al. 2005; Yian et al. 2005). The mean CS in the injured shoulder (male 84, female 73) was in both genders 10 points less than the normal value. The difference from normal values may reveal a tendency not to recover fully to the normal level after surgery.

Our study’s mean value for DASH score (14) did not differ from the normative score (10.1) (Hunsaker et al. 2002). The minimal detectable change (MDC) in DASH score is 10.5 (Roy et al. 2009). Thus, only values more than 21 in DASH score would have indicated deterioration from normal.

The reconstructive surgery does not always ensure AC joint stability. Almost half our patients suffered clinical AC joint instability after surgery. Tunnel widening may ruin stability by preventing tendon-to-bone healing. We used PLLA (poly-l-lactide acid) and PEEK (polyetheretherketone) tenodesis screws. Tunnel widening occurred similarly with both screws, with no apparent relation to screw material.

Tauber et al. (2007; 2009) and Yoo et al. (2010) have published results of chronic AC joint dislocation treatment with an autogenous semitendinosus graft. Their surgical techniques were slightly different from those we used, but their objective was similar: reconstruct the CC ligaments anatomically. Their results were encouraging in relation to functional recovery, pain-relief, patient satisfaction, and radiological findings.

Results of surgery in chronic AC joint dislocation are inferior to those in acute surgery. Von Heideken et al. (2013) compared Rockwood type-V injury which was treated acutely (within 4 weeks) with a hook plate or after a delay (after 4 months) by a modified Weaver-Dunn procedure. The acute group scored better in function, disability, pain, and satisfaction. The objectives in acute and in delayed surgery are identical: to stabilize the AC joint, restore function, and eliminate pain. These fundamental aims seem far more difficult to achieve in delayed surgery. Insufficiency of stabilizing structures, altered anatomical relations, high torsional forces in the lateral clavicle, and the presence of tunnel widening and osteolysis aggravate the site of the surgery and may prevent the success of stabilization.

We noted a high rate of complications. In almost half the patients, the AC joint was clinically unstable. It is comprehensible that the additional AC ligament reinforcement should secure the stability. Some cadaveric studies have reported encouraging results in clavicle stability after AC ligament reconstruction (Gonzalez-Lomas et al. 2010; Michlitsch et al. 2010). In a recent randomized comparative study (semitendinosus graft vs. synthetic ligament) was found lateral clavicle osteolysis in 95% of patients (Fauci et al. 2013). In our study, tunnel widening and lateral clavicle osteolysis were also remarkably common. Failure to stabilize the AC joint may be a consequence of unsatisfactory tendon-bone integration and graft incorporation. In many patients, the osteolysis affected the lateral clavicle as far as to the drill holes, causing graft loosening and instability. In only 1 patient was the osteolysis visible in the preoperative radiographs. The surgery-induced osteolysis may have been the result of a disturbance in the bone-blood circulation. Surgical dissection, clavicle drilling, or tenodesis screws may disturb blood perfusion in bone, leading to significant osteolysis. Despite the numerous complications, the patient satisfaction in surgery was surprisingly high. It would seem as complications have only minor negative effect on daily life.

Some studies discuss the arterial supply of the clavicle. Knudsen et al. (1989) found that the primary supply of the clavicle is periosteal through numerous Volkmann canals into the cortex. They found no well-developed nutrient artery. Havet et al. (2008) also found periosteal vascularisation to be the main blood supply to the clavicle. They also noticed nutrient foramina at the lateral end of the clavicle in all cases. One theory is that arterial supply to the clavicle is by the nutrient artery. Fischer and Carret (1978) found a branch of the suprascapular artery that penetrated into the clavicle via the foramina at the level of the middle-lateral third union. Certainly, some of the circulation comes also via muscle attachments. Because of chronic dislocation and stiffness of the soft tissue around the clavicle, the detachment and mobilization has to be thorough. During this detachment, the arterial supply (periosteal, nutrient artery, and muscle attachments) may undergo damage, resulting surprisingly fast in dissolution of the bone mineral, cyst formation, and finally osteolysis.

During follow-up, fractures of the coracoid process and clavicle were common. All fractures in the clavicle were in the medial drill hole; in only 1 patient did the fracture emerge due to a new injury. In 2 patients, fracture of the coracoid process was associated with a radiological cranial dislocation of the clavicle. We think that non-absorbable sutures may cause the coracoid fractures by gradually stressing or cutting the bone as suggested previously (Kippe et al. 2009). Non-absorbable sutures may offer some extra support to the tendon grafts during the healing period. When risk for stress fracture in the coracoid process is as high as in our study, it might be reasonable to avoid such sutures.

In our surgical method, the missing CC ligaments were reconstructed with autogenous tendon grafts. Non-absorbable sutures provided strength for this reconstruction. Some biomechanical studies discuss the importance of AC and CC ligaments. Urist (1946) found in his cadaver study that when CC ligaments are transected, the intact AC ligament can prevent joint dislocation. He also demonstrated how the gradual sectioning of AC and CC ligaments and muscle attachments leads to complete disarticulation. The AC ligament acts as the primary structure to prevent posterior displacement of the clavicle. The AC ligament also plays an important role in preventing superior displacement. Fukuda et al. (1986) noticed that when the clavicle is pulled upward, the first structure to resist the dislocation is the AC ligament. They discovered that the AC ligament produces two-thirds of the restraining forces against superior displacement by a lesser amount of displacement and induced load. By larger displacement and induced load, the conoid ligament played the major role. We think that in delayed surgery, the AC ligament should also be reconstructed with the tendon graft. AC ligaments are short, and in reconstruction this structure should be imitated (Stine and Vangsness 2009). Clavicular and acromial graft attachments to bone should be as close as possible to the superior and posterior part of the AC joint line.

Our study has certain limitations. As we could not examine all the patients treated with tendon grafts, some selection bias may have occurred. Surgery was not entirely standardized during the study period. Some minor modifications in surgical technique occurred over the course of time. We believe, however, that this had no major impact on results. Reconstructive surgery on CC ligaments is a demanding procedure; hence, results of surgery may be better in those patients treated in the later part of the series. The study was retrospective, leaving us unsure of the precise level of function or disability before the reconstructive surgery.

In future, what would be valuable is to compare long-term results of reconstructive surgery and nonoperative treatment in chronic AC joint dislocation. We should also determine the accurate mechanism and cause of osteolysis in delayed surgery to prevent the undesirable consequences of this complication.

## Conclusion

The aim of reconstructive surgery is to eliminate pain and discomfort by stabilizing the AC joint and maintaining, or even improving, shoulder function. Despite the surgery, almost half the AC joints failed to stabilize and also the shoulder function was inferior compared to uninjured side. Tunnel widening and lateral clavicle osteolysis were common and might ruin the results. Anatomic reconstruction of CC ligaments showed a moderate subjective outcome at the 4-year follow-up. Most patients were satisfied to the results of surgery, although radiological findings were unsatisfactory. Because of the importance of the AC ligament for clavicular stability, we recommend repairing the superior and posterior portion of the AC ligament in delayed surgery.
